# Role of corpus callosum in sleep spindle synchronization and coupling with slow waves

**DOI:** 10.1093/braincomms/fcab108

**Published:** 2021-05-25

**Authors:** Giulio Bernardi, Giulia Avvenuti, Jacinthe Cataldi, Simona Lattanzi, Emiliano Ricciardi, Gabriele Polonara, Mauro Silvestrini, Francesca Siclari, Mara Fabri, Michele Bellesi

**Affiliations:** Molecular Mind Laboratory, IMT School for Advanced Studies Lucca, Lucca, Italy; Molecular Mind Laboratory, IMT School for Advanced Studies Lucca, Lucca, Italy; Center for Investigation and Research on Sleep, Lausanne University Hospital, Lausanne 1011, Switzerland; Department of Experimental and Clinical Medicine, Marche Polytechnic University, Ancona 60126, Italy; Molecular Mind Laboratory, IMT School for Advanced Studies Lucca, Lucca, Italy; Department of Odontostomatologic and Specialized Clinical Sciences, Marche Polytechnic University, Ancona 60126, Italy; Department of Experimental and Clinical Medicine, Marche Polytechnic University, Ancona 60126, Italy; Center for Investigation and Research on Sleep, Lausanne University Hospital, Lausanne 1011, Switzerland; Department of Experimental and Clinical Medicine, Marche Polytechnic University, Ancona 60126, Italy; School of Bioscience and Veterinary Medicine, University of Camerino, Camerino 62032, Italy; School of Physiology, Pharmacology & Neuroscience, University of Bristol, Bristol BS8 1TD, UK

**Keywords:** spindle, non-REM, sleep, corpus callosum, connectivity

## Abstract

Sleep spindles of non-REM sleep are transient, waxing-and-waning 10–16 Hz EEG oscillations, whose cortical synchronization depends on the engagement of thalamo-cortical loops. However, previous studies in animal models lacking the corpus callosum due to agenesis or total callosotomy and in humans with agenesis of the corpus callosum suggested that cortico-cortical connections may also have a relevant role in cortical (inter-hemispheric) spindle synchronization. Yet, most of these works did not provide direct quantitative analyses to support their observations. By studying a rare sample of callosotomized, split-brain patients, we recently demonstrated that the total resection of the corpus callosum is associated with a significant reduction in the inter-hemispheric propagation of non-REM slow waves. Interestingly, sleep spindles are often temporally and spatially grouped around slow waves (0.5–4 Hz), and this coordination is thought to have an important role in sleep-dependent learning and memory consolidation. Given these premises, here we set out to investigate whether total callosotomy may affect the generation and spreading of sleep spindles, as well as their coupling with sleep slow waves. To this aim, we analysed overnight high-density EEG recordings (256 electrodes) collected in five patients who underwent total callosotomy due to drug-resistant epilepsy (age 40–53, two females), three non-callosotomized neurological patients (age 44–66, two females), and in a sample of 24 healthy adult control subjects (age 20–47, 13 females). Individual sleep spindles were automatically detected using a validated algorithm and their properties and topographic distributions were computed. All analyses were performed with and without a regression-based adjustment accounting for inter-subject age differences. The comparison between callosotomized patients and healthy subjects did not reveal systematic variations in spindle density, amplitude or frequency. However, callosotomized patients were characterized by a reduced spindle duration, which could represent the result of a faster desynchronization of spindle activity across cortical areas of the two hemispheres. In contrast with our previous findings regarding sleep slow waves, we failed to detect in callosotomized patients any clear, systematic change in the inter-hemispheric synchronization of sleep spindles. In line with this, callosotomized patients were characterized by a reduced extension of the spatial association between temporally coupled spindles and slow waves. Our findings are consistent with a dependence of spindles on thalamo-cortical rather than cortico-cortical connections in humans, but also revealed that, despite their temporal association, slow waves and spindles are independently regulated in terms of topographic expression.

## Graphical Abstract

**Graphical Abstract:**
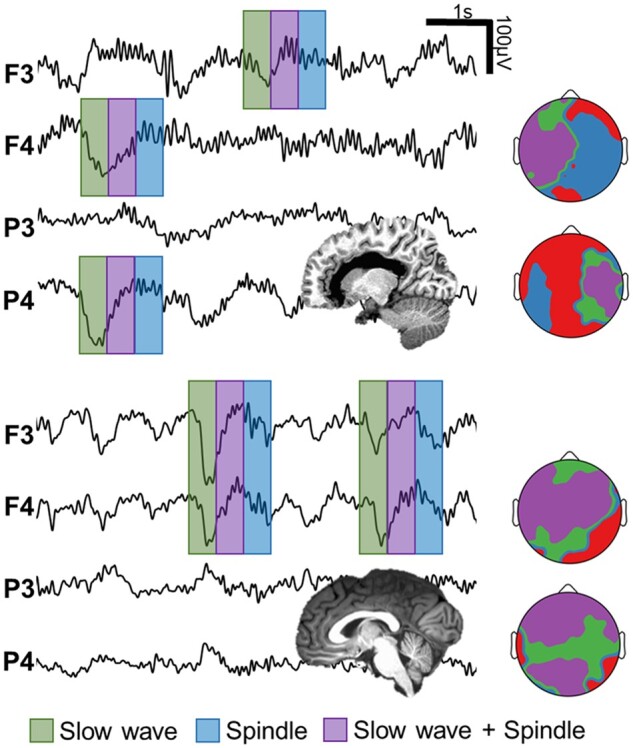

**Abbreviated summary**

Bernardi et al*.* report that complete resection of the corpus callosum does not affect the interhemispheric synchronization of sleep spindles, while it significantly reduces the spatial association between spindles and temporally coupled slow waves. These results are consistent with the dependence of spindle synchronization on thalamocortical rather than cortico-cortical connections.

## Introduction

Sleep spindles are transient waxing-and-waning 10–16 Hz electroencephalographic (EEG) oscillations that represent a typical hallmark of non-rapid eye movement (non-REM) sleep in humans.^1^ Two types of spindles are often described based on their frequency and topographic distribution: fast spindles, with frequency greater than ∼13 Hz and characterized by a centro-parietal peak of activity, and slow spindles, with frequency lower than ∼13 Hz and a typical peak in bilateral frontal electrodes. The two-spindle types may often co-occur, with fast parietal spindle activity slightly preceding in time the appearance of the slower frontal activity.^2^^,^^3^ While the two types of spindles appear to have partially different origin and regulation,^4^ they both are thought to be generated in the thalamus as a result of interactions between inhibitory, GABAergic neurons of the thalamic reticular nucleus and thalamocortical, glutamatergic relay cells,^5–7^ which mediate the synchronization of sleep spindles at cortical level.^8^ Spindles promote memory consolidation through calcium-dependent synaptic plasticity and the grouping of hippocampal sharp-wave ripples (80–200 Hz) that accompany the reactivation of cell assemblies engaged during previous wake-dependent learning.^9^^,^^10^ Accordingly, changes in spindle activity have been observed during non-REM sleep following declarative learning and acquisition of procedural motor skills.^11–14^ Moreover, learning-related spindle changes were often found to especially affect specific, spatially circumscribed, task-related cortical areas.^15^^,^^16^

While sleep spindles are generated within and by the thalamus, the cortex plays an important role in triggering, synchronizing and terminating sleep spindles.^17–19^ In line with this, spindles usually occur in association with cortically generated sleep slow waves (0.5–4 Hz).^20^^,^^21^ In particular, the *slow oscillation* (<1.5 Hz) of cortical neurons between periods of neuronal firing (*up-state*) and silence (*down-state*), which underlies the generation of EEG slow waves, has been suggested to drive the thalamic generation of sleep spindles, so that the latter most commonly appear in *up-states* surrounding *down-states*. Given that slow waves travel at cortical level through cortico-cortical connections^22^^,^^23^ and group spindles in a topographically restricted fashion,^24^^,^^25^ they could be able to modulate the expression of spindles within specific networks in order to finely regulate sleep-dependent brain plasticity.^26^

By studying a rare group of *split-brain* patients, who underwent total resection of the corpus callosum (CC), we recently demonstrated that inter-hemispheric connections are crucial for the propagation of slow waves across the two brain hemispheres.^27^ In fact, in these particular patients, slow waves tended to remain circumscribed within the hemisphere in which they originated, and the few slow waves that showed a bilateral involvement presented a slower, less efficient inter-hemispheric spreading. These results provided a direct demonstration of the strong dependence of slow waves on cortico-cortical connections for their propagation and inter-regional synchronization. Whether, and to which extent, spindle synchronization may similarly depend on cortico-cortical connections is however less clear. Previous observations in callosotomized cats^28^ and case studies in patients with agenesis of the CC described an increased occurrence of asynchronous inter-hemispheric spindles, although such a variation was not explicitly quantified.^29–32^ Moreover, cortical inactivation has been associated with a regional desynchronization of sleep spindles during natural sleep in cats^18^ (but see Contreras et al.^33^). Based on these few reports, we hypothesized that the loss of cortico-cortical inter-hemispheric connectivity could affect spindle synchronization and/or the spatio-temporal association between slow waves and spindles. In order to test these hypotheses, overnight high-density (hd-)EEG recordings of adult split-brain patients and of control subjects with an intact CC were analysed using a validated spindle detection algorithm and methods for characterizing the topographic expression of individual sleep spindles.

## Materials and methods

### Participants

Overnight hd-EEG recordings (256 electrodes, 500 Hz sampling rate; EGI) were performed at the Neurological Unit of the Marche Polytechnic University (Ancona, Italy) in five epileptic in-patients who underwent total resection of the CC [callosotomized patients (CPs); age range 40–53, two females]. All recordings were initiated at the usual bedtime of each participant and interrupted at ∼7 AM. Three non-callosotomized neurological in-patients [non-callosotomized patients (NPs); age range 44–66, two females] were also studied under the same experimental conditions. One of these patients was diagnosed with symptomatic generalized epilepsy due to viral meningoencephalitis occurred in infancy (this subject, indicated as NP03, is marked using a distinctive colour in figures). All the NPs had no diagnoses of any other comorbidities affecting brain function at the time of the study. One callosotomized patient (CP01) reported a previous diagnosis of mild, untreated obstructive sleep apnoea syndrome. None of the other patients (CP02–05 and NP01–03) had diagnosis of sleep disturbances at the time of the study. An additional control group of 24 healthy adult volunteers [healthy subjects (HSs); age range 20–47, 13 females] was studied with the same hd-EEG recording system at the Lausanne University Hospital, Switzerland. Prior to their inclusion into the study, HS group individuals underwent a clinical interview to exclude a history of sleep, medical and psychiatric disorders. None of the HSs was taking any medication at the time of the study. Relevant data on demographic and clinical characteristics of study participants has been detailed in our previous work^27^ and is summarized in Tables 1 and 2. The study procedures were conducted under clinical research protocols approved by the local ethical committees and in accordance with the guidelines of the Declaration of Helsinki. Written informed consent was obtained from all participants.

**Table 1:** Demographic and sleep characteristics

	CP01	CP02	CP03	CP04	CP05	NP01	NP02	NP03	HS (*n* = 24)
Age, years	53	40	47	45	42	45	66	44	27 ± 6
Gender	M	F	F	M	M	F	F	M	13F
Age at Surgery 1, years	30	16	25	14	18	–	–	–	–
Age at Surgery 2, years	45	17	26	22	19	–	–	–	–
Questionnaires									
PSQI	12	2	4	5	9	11	4	12	3.1 ± 1.5
ESS	23	4	2	0	13	7	18	1	5.9 ± 2.1
HOQ	59	44	43	61	54	52	34	45	51.8 ± 6.7
EHI	RH	RH	RH	RH	RH	RH	RH	RH	20RH
Sleep structure									
Sleep latency, min	20.5	29.5	23.5	38.5	34.0	6.0	6.5	4.0	15.4 ± 16.9
Total sleep time, min	244.5	243.5	185.5	254.0	120.5	278.0	251.5	118.5	256.6 ± 30.6
Sleep efficiency, %	81.4	81.0	61.7	84.5	40.1	92.5	83.7	39.4	85.5 ± 10.2
WASO, min	36	15.5	66.0	4.5	129.5	11.0	24.5	157.5	20.0 ± 15.3
N1 sleep, %	0.8	7.0	14.8	9.6	24.5	3.1	8.5	19.4	5.1 ± 5.0
N2 sleep, %	–	–	–	–	80.9	63.5	66.0	59.1	57.7 ± 10.4
N3 sleep, %	–	–	–	–	19.1	9.2	15.7	19.4	27.1 ± 7.5
NREM (N2/N3) sleep, %	100.0	88.7	100.0	100.0	100.0	72.7	81.7	78.5	84.8 ± 6.0
REM sleep, %	0.0	11.3	0.0	0.0	0.0	27.3	18.3	21.5	15.2 ± 6.0

For patients in CP and NP groups, demographic characteristics, questionnaires, and sleep macro-architecture are presented separately for each subject. For CP patients, the resection of the corpus callosum occurred in two distinct surgical interventions, for each of which the age of the patients is reported. For the HS group, the values are reported as group mean ± standard deviation. Sleep stage percentages are expressed with respect to total sleep time (TST). CP, callosotomized patients; EHI, Edinburgh Handedness Inventory; ESS, Epworth Sleepiness Scale; HOQ, Horne-Őstberg Questionnaire; HS, healthy subjects; NP, non-callosotomized patients; PSG, polysomnography; PSQI, Pittsburg Sleep Quality Index; WASO, wake after sleep onset.

**Table 2:** Clinical diagnosis and medications of studied patients

	Diagnosed pathology	Current medications
CP01	Lennox–Gastaut Syndrome	Carbamazepine, Phenytoin sodium, Phenobarbital
CP02	Drug-resistant epilepsy	Carbamazepine, Levetiracetam, Sodium valproate
CP03	Early Infantile Epileptic Encephalopathy	Levosulpiride, Oxcarbazepine, Phenobarbital, Risperidone
CP04	Lennox–Gastaut Syndrome	Clobazam, Lacosamide, Phenobarbital, Rosuvastatin, Vigabatrin
CP05	Drug-resistant epilepsy	Carbamazepine, Clonazepam, Diazepam, Omeprazole, Phenobarbital
NP01	Generalized Anxiety Disorder	None
NP02	Lumbar spinal stenosis	Cholecalciferol, Esomeprazole, Lisinopril, Mometasone
NP03	Epilepsy (viral meningoencephalitis in infancy)	Enalapril+Lercanidipine, Lacosamide, Oxcarbazepine, Sodium valproate, Ursodeoxycholic acid

CP, callosotomized patients; NP, non-callosotomized patients.

### Data preprocessing

For scoring purposes, four electrodes were used to monitor horizontal and vertical eye movements (electrooculography), while electrodes located in the chin-cheek region were used to evaluate muscular activity (EMG). Sleep scoring was performed over 30-s epochs according to the criteria from the American Academy of Sleep Medicine scoring manual.^34^ Two operators took care in marking periods containing large artefacts, arousals and non-physiological activity.^27^ Of note, patients CP01–CP04 and NP03 presented relatively frequent periods of non-physiological activity during their sleep, which limited the possibility to accurately distinguish between N2 and N3 sleep. Such events were less common in patient CP05 (this subject is thus marked using a distinctive colour in figures).

Given that CPs presented relatively low sleep quality with frequent awakenings, especially in the second part of the night, we extracted and analysed only the first 5 h of each recording, starting from the time of ‘lights-off’.^27^ For all patients, recordings were band-pass filtered between 0.1 and 45 Hz. Then, overnight recordings were divided into 30-s epochs, while wake resting-state recordings were divided into 4-s epochs. Bad channels and epochs were identified and rejected through visual inspection in NetStation 5.3 (EGI). An Independent Component Analysis procedure was used to reduce residual ocular, muscular and electrocardiograph artefacts (EEGLAB toolbox^35^). Finally, rejected bad channels were interpolated using spherical splines. A similar procedure was used to pre-process the sleep data of healthy control subjects, as detailed in previous work.^36^ In all subjects, only N2 and N3 sleep data-segments free from artefacts, arousals and non-physiological activity were eventually analysed.

### Spindle detection

The detection of sleep spindles was performed using the open-source *sleep wave analysis toolbox.*^37^^,^^38^ The analysis proceeded along three key stages.^38^ First, the data were re-referenced to average-reference and the average EEG activity of nine, equidistant, non-overlapping scalp regions was calculated. The regions included a mean of 12 electrodes (±2.8), for a total of 104 channels. This particular step has the aim of reducing the number of channels on which spindle detection runs while also improving signal-to-noise ratio of the spindle events. In the second stage, a validated spindle detection algorithm was applied to each of the nine time-series.^39^^,^^40^ Specifically, the time-series were filtered using a b-spline wavelet,^41^ and obtained values were squared and smoothed using a 100-ms sliding window. All cases in which the obtained signal power crossed a threshold corresponding to four times the median absolute deviation from the median of each sleep epoch were regarded as potential spindles. The actual starts and ends of candidate spindles were then estimated based on the crossing times of a second, low-threshold at 2 median absolute deviations. Only events with a duration comprised between 0.3 and 3 s, and whose ratios between mean signal power (Welch’s method) in the 10–16 Hz (sigma) range and mean power in the neighbouring 8–10 Hz and 16–18 Hz ranges were greater than 3, were retained for further evaluation. The power-ratio criterion was applied in order to ensure some specificity of the transient power increases within the spindle range, thus excluding events associated with broadband power increases, such as microarousals. Spindles detected in distinct scalp regions that showed a relative overlap in time (of any length) were considered as single events and combined. Finally, in the third stage, all the individual electrodes were re-examined to determine the actual scalp distribution of each detected spindle. In particular, the Welch’s method (Hamming window with length equal to spindle duration) was used to compute the signal spectral power for each spindle and channel, and two main parameters were then obtained: the power in the sigma range (hereinafter referred to as scalp ‘involvement’) and the ratio of the mean power in the sigma range over the mean power in the neighbouring ranges (8–10 Hz and 16–18 Hz). Individual electrodes showing a power-ratio greater than 3 were considered as recruited in a specific spindle event, and thus used to build a scalp ‘recruitment’ map. The ‘probabilistic recruitment’ was defined as the overall probability for each electrode to be recruited during sleep spindles. Of note, both involvement and recruitment maps were computed for 191 ‘internal’ electrodes, after exclusion of channels located on the neck and face regions. This approach was aimed at minimizing the risk of including artifactual spindle-like activity.

### Principal component analysis

Using a principal component analysis (PCA), we recently showed that 95% of the variance related to slow-wave scalp involvement is explained by three principal components^42^ (PCs), with maxima located in the centro-frontal area, anterior or posterior areas and left or right hemispheres. We then demonstrated that the variance explained by the latter of the three components increases significantly in CPs relative to HSs, reflecting an increased occurrence of uni-hemispheric slow waves.^27^ In this respect, the PCA may offer a simple, data-driven method for the identification of significant variations in the spatial distribution of specific EEG sleep events. Given these premises, here we applied a similar approach for the analysis of the topographic distribution of sleep spindles, to specifically investigate whether uni-hemispheric PCs increased at the expense of symmetrical PCs. To this aim, the channel-wise sigma-range power values obtained for each detected spindle were normalized through *z*-scoring (across electrodes) and entered in a PCA. This analysis was performed separately for HS, NP and CP groups. Based on an initial inspection of the obtained components, we retained and included in subsequent analyses only the first six PCs of each subject. This choice was driven by two main motivations: these six components were relatively stable across subjects and groups, and, in most subjects, each component explained at least 5% of the total variance. The PCs-space of each subject was rotated into a common, reference-PCs-space using the Procrustes transformation.^43^^,^^44^ The reference-space was selected by iteratively applying the transformation over pairs of subjects of the HS group and then identifying the coordinate system (i.e. the subject) presenting the smallest distance with respect to the coordinate systems of all tested subjects.^44^ Finally, the Procrustes transformation was applied to remap the original PCs-space of each subject (including the patients), into the new reference-PCs-space. This procedure allowed us to directly compare the PCs, and their explained variances, across individuals.^27^

### Inter-hemispheric recruitment asymmetry

As described in our previous work, an asymmetry index was computed to quantify the relative distribution of electrodes recruited in the same sleep spindle across the two hemispheres.^27^ In particular, the recruitment asymmetry index was defined as the number of channels in the hemisphere with less recruited electrodes, divided by the total number of recruited channels (%). The index was then expressed as 100 minus the obtained value, so that 50% indicates a symmetric distribution, while 0% indicates a unilateral event. The same parameter was also computed separately for the slowest (lowest 25th frequency percentile) and the fastest (highest 25th frequency percentile) sleep spindles. Spindles were classified using an individual threshold based on percentiles rather than a fixed arbitrary threshold (e.g. 13 Hz) for two main reasons. First, the use of an individualized threshold allowed us to take into account variations in spindle frequency within and across subjects. Second, by excluding spindles with an intermediate frequency we expected to maximize potential differences in the effects of callosotomy on the two-spindle sub-types. A globality index, reflecting the percentage of recruited electrodes with respect to the total number of channels was also calculated.

### Temporal and spatial overlap with sleep slow waves

Sleep spindles are known to commonly occur in association with sleep slow waves.^45^ Thus, here we investigated whether callosotomy affected in any way the temporal and/or spatial aspects of such coupling. To this aim, slow waves were detected, and their relative scalp propagations were computed as described in previous work.^27^ In brief, the 0.5–45 Hz bandpass-filtered EEG signal was re-referenced to mastoid-reference and a negative-going signal envelope was calculated by selecting the fifth most negative sample across the 191 internal electrodes.^3^^,^^37^ The obtained signal-envelope was zero-mean recentered (0.5–40 Hz broadband filter) and potential slow waves were identified as negative half-waves comprised between two consecutive signal zero-crossings.^3^^,^^46^ Only half-waves with a duration comprised between 0.25 and 1.0 s were retained for further analyses. No amplitude thresholds were applied. Then, for each detected slow wave, the pattern of propagation was calculated by determining the topographic distribution of relative delays in the local maximum negative peak, representing the moment of maximal regional recruitment.^22^ A ‘likeness constraint’ method^47^ was used to discard channels in which the negative wave was excessively dissimilar from a ‘prototype’ slow wave, defined as the wave with the largest negative peak at the reference-peak timing across all channels. Specifically, we calculated the cross-correlation between the instantaneous phases (estimated using the Hilbert transform) of the prototype wave and of all other EEG signals in a symmetrical 300 ms time-window centred on the reference peak. The 25th percentile of the distribution of the maximal cross-correlation values was then used as a threshold to exclude events dissimilar from the prototype wave. A clusterization procedure was applied to the latencies of all remaining local peaks to exclude potential spatiotemporal propagation gaps (maximum allowed interval between neighbour electrodes was set to 10 ms). Finally, the propagation cluster including the prototype wave was identified and the final delay map was extracted. For the aims of the present study, the delay maps were obtained to derive topographic recruitment maps for all sleep slow waves.

Spindles that occurred in a time-window extending from 1 s before the first zero-cross to 1 s after the second zero-cross of a detected slow wave, and for which a spatial overlap with the same slow wave was also observed in at least one electrode were considered as coupled with a slow wave event. In particular, the spatial overlap was determined using the spindle and the slow-wave recruitment maps. For each spindle associated with a slow wave, we then computed the overall overlap extent index, expressed as the percentage of overlapping electrodes with respect to the total number of electrodes recruited in the spindle event.

### Statistical analyses

For each parameter of interest, the five CPs and the three NPs (Ancona dataset) were compared with the control group of healthy adult subjects (HS; Lausanne dataset). Specifically, for each patient, the relative *z*-score and corresponding *P*-value were computed with respect to the distribution represented by the HS group. A Bonferroni correction was applied to account for multiple comparisons across tested subjects and related hypotheses. Effects were regarded as significant only when a corrected *P *<* *0.05 was observed in each of the five CPs and in none of the three NPs (Table 3 summarizes group- and subject-level statistics for each performed comparison). Analyses were repeated after regression-based adjustment of values to account for inter-subject age differences. When not differently specified in text, adjustments for age did not significantly affect the results. The same statistical approaches were used in our previous work based on the assumption that the resection of the CC should represent a change of such a magnitude that strong, consistent variations should be expected in all the subjects who underwent the procedure.^27^

**Table 3:** Summary of statistics related to comparisons between patients and healthy subjects

		Healthy subjects (*N* = 24)					Non-call. patients			Callosotomized patients				
Analysis	Parameter	KS test	MEAN	SD	PRC. 2.5	PRC. 97.5	NP01	NP02	NP03	CP01	CP02	CP03	CP04	CP05
Properties	Density	0.703	15.8	2.0	12.7	19.8	16.4	14.2	6.6*	4.9*	8.3*	9.6*	**11.8**	18.9
	Amplitude	0.900	29.8	5.9	21.1	39.4	**40.8**	23.7	26.3	46.0*	52.9*	61.5*	63.7*	**43.0**
	Frequency	0.967	12.8	0.3	12.3	13.4	12.4	12.5	12.6	12.8	12.9	**12.0**	12.5	12.3
	Duration	0.446	0.9	0.1	0.8	1.2	0.8	0.8	0.5*	0.6*	0.6*	0.5*	0.5*	0.6*
Involv. PCA	PC1	0.552	35.3	7.0	23.1	49.0	30.4	29.4	**12.5**	25.8	**17.6**	**12.7**	**18.2**	32.6
	PC2	0.9	28.2	6.7	16.1	40.6	34.1	25.5	32.1	17.0	19.5	24.1	35.9	20.3
	PC3	0.039	11.6	4.0	7.5	27.1	8.2	13.1	13.9	14.4	13.8	8.4	11.2	11.1
	PC4	0.794	11.0	2.5	6.5	17.2	12.3	13.6	15.8	16.1	16.8	22.6*	15.4	13.1
	PC5	0.846	7.4	2.2	4.5	13.7	8.5	11.3	11.4	14.2	24.0*	12.3	10.9	11.5
	PC6	0.617	6.5	2.0	4.3	12.8	6.5	7.2	14.4*	12.5	8.4	19.9*	8.3	11.4
Involvement distribution.	Globality	0.834	45.8	6.0	33.1	58.1	50.8	46.8	35.2	**32.1**	**32.6**	27.5*	34.2	49.6
	Asymmetry	0.834	38.8	1.5	35.7	41.2	39.6	36.9	36.4	32.8*	33.6*	31.0*	34.5*	40.9
	Asym. Slow	0.998	38.0	1.9	34.9	41.5	38.6	35.8	37.8	34.8	**33.4**	32.6*	35.2	41.2
	Asym. Fast	0.900	39.3	1.4	36.7	42.2	41.0	38.3	**35.9**	32.9*	33.4*	29.0*	35.0*	40.4
Association with SWs	Probability	0.865	60.0	11.1	34.9	76.6	54.9	61.9	52.1	70.5	64.5	69.9	42.8	51.9
	Sp. Overlap	0.939	54.9	3.1	48.9	60.6	56.2	**47.1**	54.6	39.0*	44.5*	37.0*	42.3*	42.6*

The first two columns indicate the analyses of interest. Columns three to seven include descriptive statistics for the healthy subjects (HS) group: *P*-value of the Kolmogorov–Smirnov test for data normality (KS Test), group level mean (Mean), standard deviation of the mean (SD), 2.5 (Prc. 2.5) and 97.5 (Prc. 97.5) percentiles of the distribution. Columns from eight to ten show the values of the parameter of interest observed in each of the three non-callosotomized patients (NP01–NP03). Columns from ten to thirteen show the values of the parameter of interest observed in each of the five callosotomized patients (CP01–CP05). Bold text indicates values that fall off the 2.5–97.5 percentiles range (*α* < 0.05).

* marks values that are significantly different from those of the HS group after Bonferroni correction. The correction was applied based on the number of tested subjects (*N* = 8). The PCA-based analysis represents an exception as in this case the correction also took into account the number of PCs that were tested.

### Data availability

Relevant data that support the findings of this study are available from the corresponding authors upon motivated request.

## Results

### Spindle characteristics

**Figure 1:**
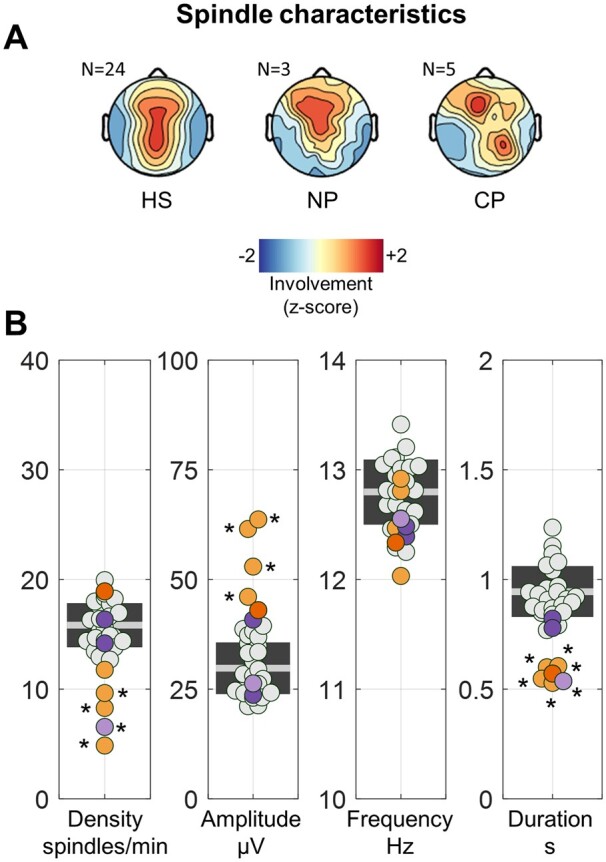
**Properties of sleep spindles.** (**A**) Scalp distribution (involvement) of spindle activity (signal power in the 10–16 Hz range) during sleep spindles. (**B**) Mean spindle density (spindles/min), amplitude (µV), and frequency, for each subject. CPs are represented with orange dots (CP05 = dark orange), NPs with purple dots (NP03 = light purple), and HSs with light-gray dots. The light-gray horizontal line represents the mean for the HSs, while the dark-gray box represents 1 SD around the mean. Values observed in each patient were compared with those of the HS group using *z*-tests and a Bonferroni adjustment was applied to correct for multiple comparisons. *P_cor._ < 0.05.

Figure 1A shows the average topographic distribution of spindle involvement, which presented a similar fronto-parietal distribution in all the three groups of subjects (CP, NP, HS). Quantitative comparisons of spindle properties across groups showed that the mean density (CP = 10.7 ± 5.2 spindle/min, range 4.9–18.9; HS = 15.8 ± 2.0 spindle/min, range 12.7–19.9), amplitude (CP = 53.4 ± 9.1 µV, range 43.0–63.7; HS = 29.8 ± 5.9 µV, range 21.1–39.4) and frequency (CP = 12.5 ± 0.4 Hz, range 12.0–12.9; HS = 12.8 ± 0.3 Hz, range 12.3–13.4) of spindles were not systematically different between CPs and HSs (Fig. 1B). Specifically, for spindle density, we found significant differences (*P*_cor._ < 0.05) for CP01 (*P*_unc._ < 0.001, |*z*| = 5.442), CP02 (*P*_unc._ < 0.001, |*z*| = 3.742), CP03 (*P*_unc._ = 0.002, |*z*| = 3.067) and NP03 (*P*_unc._ < 0.001, |*z*| = 4.601). However, the effects remained significant only for CP01 and CP03 after controlling for between-subjects age differences. For spindle amplitude, we found significant differences (*P*_cor._ < 0.05) in four of the five callosotomized patients (CP01–04; *P*_unc._ < 0.006, |*z*| ≥ 2.758). No significant effects were found for spindle frequency either with (*P*_unc._ > 0.01, |*z*| ≤ 2.581) or without (*P*_unc._ > 0.03, |*z*| ≤ 2.169) adjustment for age differences. Finally, we found that all epileptic patients, including control subject NP03, had reduced spindle duration with respect to the HS group (*P*_cor._ < 0.05, |*z*| ≥ 2.918; CP = 0.57 ± 0.03 s, range 0.53–0.61; HS = 0.94 ± 0.1 s, range 0.77–1.24). After adjustment for age differences the effect remained significant in all CPs (*P*_cor._ < 0.05, |*z*| ≥ 2.958) but not in NP03 (*P*_unc._ = 0.03, |*z*| = 2.182). A visual inspection of spindle-duration distribution histograms further showed that ∼90% of spindles in CPs had duration <0.9 s, while only ∼55% of spindles fell within the same range in HSs. These results may indicate an altered capacity of the callosotomized brain to sustain long-lasting spindles.

In order to facilitate comparison with previous studies, properties of sleep spindles independently detected in individual electrodes have been included in Supplementary Fig. 1.

### Spindle involvement

**Figure 2:**
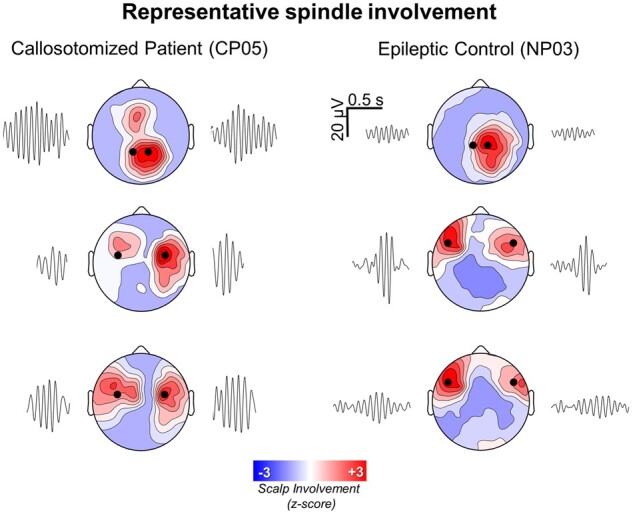
**Representative spindle involvement patterns.** Representative spindle involvement patterns are shown for a callosotomized (CP05) and a non-callosotomized (NP03) epileptic patient. Each topographic plot represents a distinct spindle event. The black dots correspond to the electrode associated with the highest signal power in the 10–16 Hz range and to the symmetric electrode across the nasion-inion axis. The wavelet filtered signal (10–16 Hz) extracted from these two electrodes is shown on the left and on the right of each topographic plot.

Visual inspection of EEG-traces revealed no obvious differences in the topographic channel recruitment of individual spindles across experimental groups (Fig. 2).

**Figure 3:**
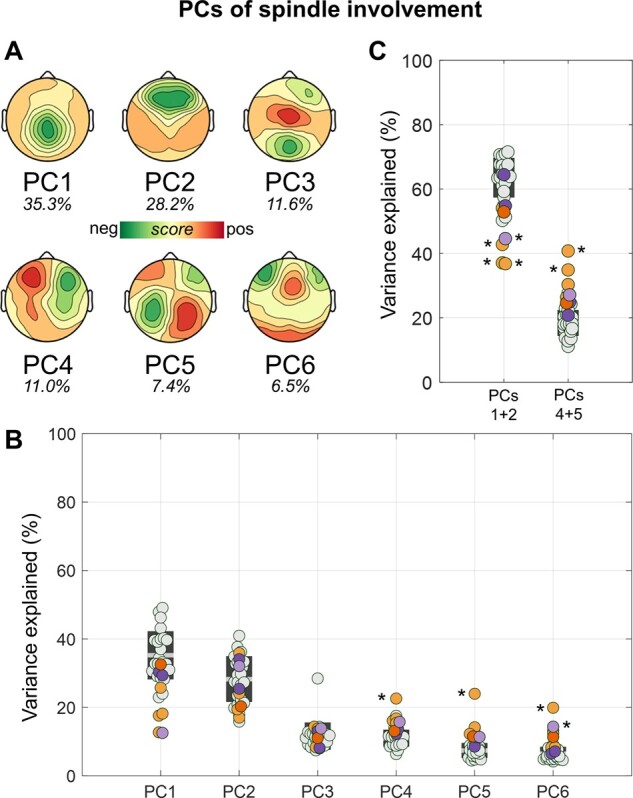
**PCA-based analysis of spindle involvement.** The involvement distribution (*z*-scored signal power computed in a time-window corresponding to the spindle event) of all spindles was entered in a PCA. Six main components explained more than 5% of the variance in most subjects. (**A**) Topographic distribution of each PC and corresponding, re-scaled explained variance (the sum of the variance of the six components is 100%). (**B**) The plot shows the variance explained by each of the six PCs in all subjects. CPs are represented with orange dots (CP05 = dark orange), NPs with purple dots (NP03 = light purple), and HSs with light-gray dots. (**C**) The same as in (**B**), but here the variance explained by PC1 and PC2, and that of PC4 and PC5 were combined. Values observed in each patient were compared with those of the HS group using *z*-tests and a Bonferroni adjustment was applied to correct for multiple comparisons. *P_cor._ < 0.05.

This observation was also quantitatively confirmed through a PCA-based comparison of spindle involvement (Fig. 3). In fact, in HSs, six PCs explained between ∼80% and ∼90% of the variance related to scalp spindle involvement, with maxima located in the medial parietal area (PC1: 35.3 ± 7.0%, of the total variance explained by the first six components), frontal areas (PC2: 28.2 ± 6.7%), occipital and frontal or central areas (PC3: 11.6 ± 4.0%), left or right frontal areas (PC4: 11.0 ± 2.5%), left or right parietal areas (PC5: 7.4 ± 2.2%), bilateral frontal or occipital and medial-frontal areas (PC6: 6.5 ± 2.0%), respectively. Similar values were obtained in the NP and in the CP groups (*P*_cor._ < 0.05; Bonferroni correction based on the number of tested subjects and PCs). Indeed, we only found an increase in the variance explained by PC4 in subject CP03 (*P*_unc._ < 0.001, |*z*| = 4.683), by PC5 in subject CP02 (*P*_unc._ < 0.001, |*z*| = 7.570) and by PC6 in subjects CP03 (*P*_unc._ < 0.001, |*z*| = 6.806) and NP03 (*P*_unc._ < 0.001, |*z*| = 4.006). Of note, for PC3, subject HS02 represented an outlier with respect to the main distribution of the HS group, but its removal did not affect the results. After adjustment for inter-subject age differences, the most relevant variations were observed for PC5, for which a significant increase in explained variance was found for CP01–03 and CP05 (*P*_unc._ < 0.001, |*z*| = 3.373), but not for CP04 (*P*_unc._ = 0.348, |*z*| = 0.938).

To account for potential false negative results due to the stringent correction for multiple comparisons, we ran an additional analysis in which we compared the groups after combining the variance of PC1 and PC2, representing the most common symmetrical PCs, and the variance of PC4 and PC5, representing asymmetrical PCs. Again, no systematic differences were found between CPs and HSs. Specifically, we found a relative decrease in the variance explained by the first two PCs in CP01, CP02 and CP03 (*P*_unc._ < 0.001, |*z*| ≥ 3.339), and a relative increase in the variance explained by the combined fourth and fifth PCs in CP02 and CP03 (*P*_unc._ < 0.004, |*z*| ≥ 2.938). For this latter comparison, a significant effect also emerged in CP01 after adjustment for age (*P*_unc._ < 0.001, |*z*| = 3.622).

### Inter-hemispheric recruitment asymmetry

**Figure 4:**
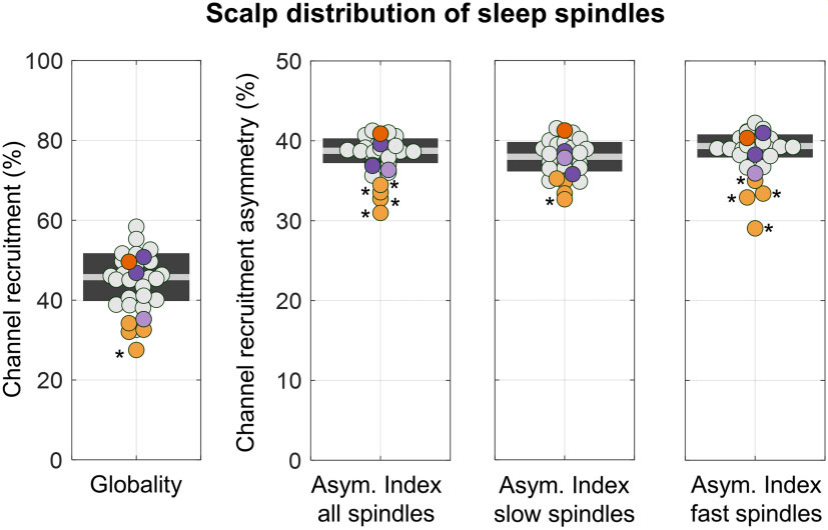
**Quantitative analysis of spindle cross-hemispheric involvement.** In the first plot, the globality score was computed as the average percentage of electrodes recruited during the spindle events. In particular, for each detected spindle, individual electrodes were considered as recruited if the ratio between the signal power in the 10–16 Hz range and the mean power in the neighbouring frequencies (8–10 Hz and 16–18 Hz) was greater than 3. The recruitment asymmetry was determined by computing the number of channels in the hemisphere with less recruited electrodes divided by the total number of recruited channels across the two hemispheres. Values close to 50% indicate a symmetric distribution, while values close to 0% indicate a unilateral wave. This parameter was computed for all detected spindles, as well as separately for the slowest (lowest 25th frequency percentile) and fastest (highest 25th frequency percentile) spindles. Values observed in each patient were compared with those of the HS group using *z*-tests and a Bonferroni adjustment was applied to correct for multiple comparisons. CPs are represented with orange dots (CP05 = dark orange), NPs with purple dots (NP03 = light purple), and HSs with light-gray dots. *P_cor._ < 0.05.

We then investigated whether callosotomy was associated with a significant change in spindle synchronization efficiency as measured at scalp level based on the proportion of involved electrodes (Fig. 4). This analysis revealed no overall differences between CPs and HSs (CP = 35.2 ± 8.4%, range 27.5–49.6; HS = 45.8 ± 6.0%, range 32.6–58.4). A significant effect was found only for subject CP03 (*P*_unc._ = 0.002, |*z*| = 3.041). Next, we examined whether the degree of inter-hemispheric asymmetry in spindle scalp distribution diverged significantly between CPs and HSs. The degree of asymmetry tended to be larger in the CP group (CP = 34.5 ± 3.8%, range 31.0–40.9; HS = 38.7 ± 1.5%, range 35.7–41.3), for which a significant difference relative to HSs was detected in four of the five patients (CP01–04; *P*_unc._ < 0.006, |*z*| ≥ 2.793). However, the difference remained significant only in CP01 (*P*_unc._ < 0.001, |*z*| = 4.422) and CP03 (*P*_unc._ < 0.001, |*z*| = 5.707) after adjustment for inter-subject age differences. The same analysis was also repeated separately for the slowest (lowest 25th frequency percentile) and the fastest (highest 25th frequency percentile) sleep spindles. For slow spindles, a significant asymmetry increase was found only in CP03 (*P*_unc._ = 0.004, |*z*| = 2.849), while for fast spindles, an inter-hemispheric asymmetry increase was identified in four of the five CPs (CP01–04; *P*_unc._ < 0.003, |*z*| ≥ 3.004). Similar results were obtained using a fixed, arbitrary threshold to define slow (<13 Hz) and fast (>13 Hz) spindles (*data not shown*).

### Coupling between sleep spindles and slow waves

**Figure 5:**
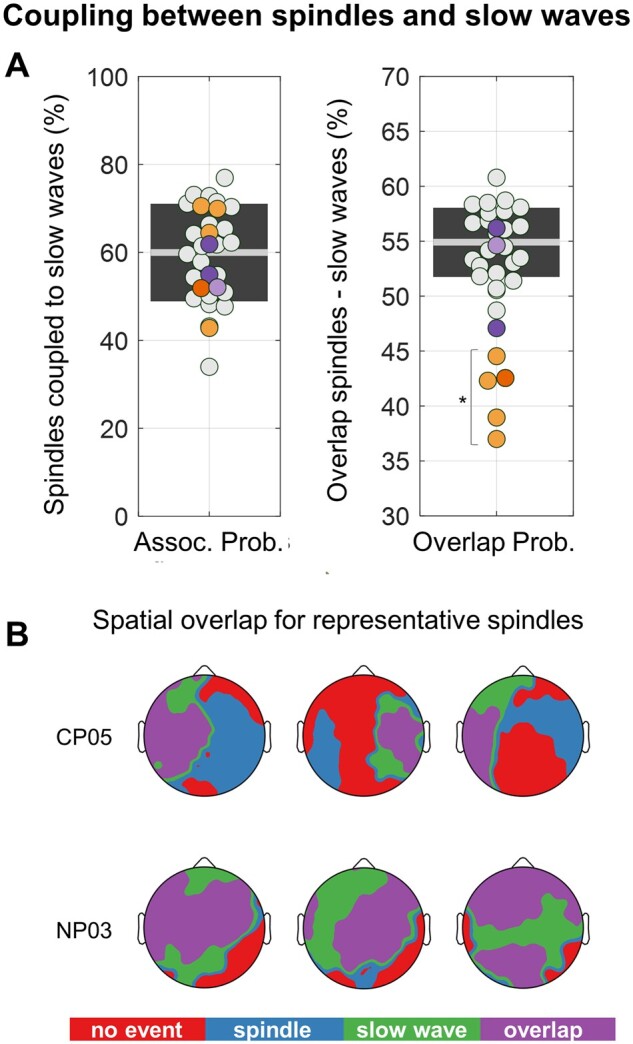
**Coupling between spindles and slow waves.** Spindles that occurred in a time-window extending from 1 s before the first zero-cross to 1 s after the second zero-cross of a slow wave, and for which a spatial overlap with the same slow wave was also observed in at least one of the 191 internal electrodes, where considered as coupled with a slow wave event. Given that slow waves appear to be affected by callosotomy, while spindles seem not or scarcely affected, we expected the coupling between these two events to be altered in callosotomized patients. (**A**) The plot on the left shows the proportion of spindles associated with a slow wave, while the plot on the right shows the proportion of electrodes presenting an overlap between spindles and slow waves with respect to the total number of electrodes recruited in spindle events. Values observed in each patient were compared with those of the HS group using *z*-tests and a Bonferroni adjustment was applied to correct for multiple comparisons. CPs are represented with orange dots (CP05 = dark orange), NPs with purple dots (NP03 = light purple), and HSs with light-gray dots. *P_cor._ < 0.05. The topographic plots in (**B**) show representative examples of the spatial overlap between spindles and slow waves in a callosotomized (CP05) and a non-callosotomized (NP03) epileptic patient.

**Figure 6:**
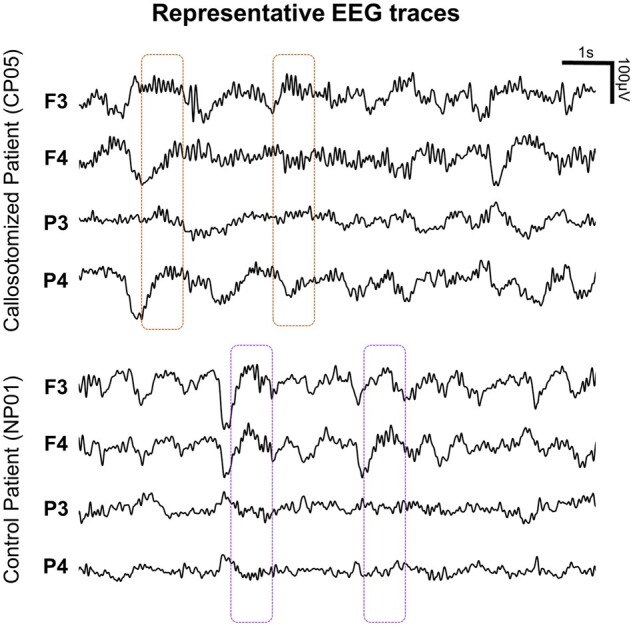
**Representative EEG traces.** Representative EEG traces (negative-down on the y-axis) containing slow waves and sleep spindles are shown for a callosotomized (CP05; top) and a control, non-callosotomized patient (NP01; bottom). Dashed boxes indicate time-windows in which sleep spindles were detected. In callosotomized subjects, spindles occurring in the down-to-up state transition of slow waves often involved both brain hemispheres even if the associated slow waves were uni-hemispheric.

Finally, given our previous work showing that callosotomy significantly affected the inter-hemispheric propagation of sleep slow waves, we here investigated whether it also altered the coupling between spindles and slow waves. First, we evaluated whether the proportion of spindles temporally and spatially associated with slow waves was significantly different between CPs and HSs (Fig. 5A). We found no differences in coupling probability across the two groups (CP = 59.9 ± 12.1%; HS = 60.0 ± 11.1%; All *P*_unc._ > 0.122, |*z*| ≤ 1.547). Similar coupling probability values were also found in the NP group (56.3 ± 5.0%). By contrast, we found that the spatial overlap between individual spindles and slow waves was significantly reduced in all CPs relative to HSs (*P*_cor._ < 0.05, |*z*| ≥ 3.294; Figs. 5B and 6; also see Supplementary Fig. 2). A similar difference was not observed in any of the NPs (*P*_unc._ > 0.013, |*z*| ≤ 2.484).

## Discussion

Taking advantage of the unique opportunity to study a rare population of split-brain patients, here we showed that, unlike slow waves, whose inter-hemispheric synchronization strongly depends on the integrity of CC,^27^ the inter-hemispheric synchronization of sleep spindles appears to be only modestly influenced by the lack of inter-hemispheric anatomical connections. Consequently, the spatial (but not the temporal) coupling between spindles and slow waves is remarkably reduced in CPs. Taken together, these results indicate that the cortical synchronization of sleep spindles is mostly independent from the integrity of inter-hemispheric connections and occurs independently from the synchronization and propagation of sleep slow waves.

### General characteristics of sleep spindles in CPs

Sleep spindles of CPs showed a similar mean frequency with respect to healthy adult subjects but tended to have a lower density and a higher amplitude. In particular, a reduced density was found in three of the CPs but also in the epileptic control patient (NP03), implying that these changes could depend on the underlying pathological conditions or their pharmacological treatments,^48^ and may thus not represent a direct consequence of callosotomy. It should be noted that spindle density in our samples is higher with respect to that reported in previous work on a large sample of healthy adult individuals (e.g. Warby et al.^40^). The main reason is that, while spindle density is commonly expressed as the number of events per unit time in a single electrode, the present analysis evaluated the occurrence of spindles across distinct regions of interest covering a large portion of all available scalp electrodes (see Supplementary Fig. 1).

Spindle amplitude was found to be relatively larger in all callosotomized subjects, although the effect reached significance only in four of the five patients. The increased amplitude in these patients may reflect a greater thalamo-cortical excitability^48^ and/or maladaptive synaptic plasticity due to the underlying pathological condition.^49^ An effect of pharmacological treatments is also possible. Indeed, previous work showed that benzodiazepines, for instance, may lead to increases in signal power between ∼10 and ∼14 Hz, as well as in spindle amplitude.^50^ A direct effect of callosotomy is instead unlikely in light of previous evidence of a positive correlation between spindle power and integrity of the CC as measured using diffusion tensor imaging^51–53^ (but see Sanchez et al.^54^). In line with this, studies in rats showed that callosotomy may lead to an increased generation of spindle bursts, probably due to the loss of a CC-mediated modulation of inhibitory interactions between homotopic regions, but only if surgery is performed within the first week of life, while no significant effects are observed afterwards.^55^^,^^56^

Finally, we found that spindle duration was significantly reduced in all epileptic subjects (including control patients NP03) relative to healthy control subjects, and that this effect remained significant only in CPs after adjustment for age. These results did not allow us to unambiguously determine whether the observed variation in spindle duration depends on the underlying pathological condition or its pharmacological treatment, or it rather reflects a direct consequence of callosotomy. Interestingly, previous evidence suggested that spindle termination may depend on cortico-thalamic feedback and on the desynchronization between cortical and thalamic structures.^19^^,^^57^ In this respect, the resection of the CC could lead to a more rapid desynchronization of spindle activity in distinct cortical areas, which may, in turn, lead to more rapid termination of the spindle sequence due to asynchronous cortico-thalamic feedback.

### Effects of callosotomy on inter-hemispheric spindle synchronization

While sleep spindles were classically described as widespread (or even ‘global’) events, their evaluation in intracranial and scalp high-density EEG recordings revealed that they actually represent mostly local and locally regulated events.^2^^,^^3^ Indeed, in a previous high-density EEG investigation, we showed that the proportion of electrodes involved in sleep spindles tend to increase during the falling asleep process, reaching maximum values comprised between 40 and 50%.^3^ Here we confirmed this observation, by showing that spindles tended to recruit, on average, 40–50% of all examined electrodes in HSs. Importantly, this percentage was not significantly affected by callosotomy, indicating that inter-hemispheric connections are not necessary for the occurrence of relatively widespread spindles. However, a more detailed evaluation of inter-hemispheric topographic spindle asymmetry did not allow us to entirely exclude a possible effect of callosotomy on inter-hemispheric spindle synchronization. In fact, we found that spindles tended to be more asymmetric in at least some of the callosotomized subjects, although this effect never reached significance in more than four of the five patients across distinct analyses. Of note, this asymmetry was especially evident for fast spindles, while slow spindles showed minimal differences between CPs and healthy control subjects. While to be considered with caution, this observation may add to previous evidence indicating that fast and slow spindles undergo distinct circadian, homeostatic, genetic and age-dependent regulation,^4^ suggesting that they may be also characterized by a different dependence on cortico-cortical connections for their synchronization. Taken together, our present results and previous observations on sleep slow waves,^27^ suggest that the effect of callosotomy on inter-hemispheric spindle synchronization is less strong and consistent with respect to the one observed for slow waves. This is coherent with a predominant role of the thalamus and of thalamo-cortical loops in generating and synchronizing sleep spindles.

### Callosotomy is associated with a regional decoupling between spindles and slow waves

While in CPs the proportion of spindles temporally coupled to slow waves was similar to that of healthy control subjects^21^^,^^58^ (i.e. ∼60%), the spatial overlap between the two EEG events was remarkably reduced. This finding likely resulted from the combined effect of a reduced inter-hemispheric spreading of sleep slow waves in conjunction with a (quasi) normal inter-hemispheric synchronization of sleep spindles in CPs. Therefore, it is possible to speculate that the regional synchronization of spindles and slow waves may rely on partially independent mechanisms. Specifically, our results suggest that slow waves may contribute to the initiation of associated spindles but are not responsible for regionally synchronizing sleep spindles during their cortical propagation. In other words, spindles could be synchronized through mechanisms that are independent from those that regulate the cortical propagation of sleep slow waves. It remains, however, unclear whether a temporally intact but spatially altered coupling between spindles and slow waves, such as the one described in the present work, could have significant consequences on experience-dependent brain plasticity and learning.^26^ Indeed, while previous work indicates that the temporal association between slow waves and spindles is essential for sleep-related memory consolidation processes (e.g. Refs.^59–62^) little attention has been directed to the role of the spatial, and in particular network-level, association between these two sleep EEG oscillations.

### Limitations

Split-brain patients represent an exceptionally rare population.^63^ In order to account for limitations related to the small sample size of patients, we performed all evaluations at single-subject level and applied strict criteria for the definition of significant group-level differences.^27^ Such strict criteria may have led to false negative results, and we cannot thus exclude a possible (though small) effect of callosotomy on spindle synchronization. Moreover, spindle-activity indices of all the epilepsy patients (CP01–CP05 and NP03) could have been affected by the underlying pathological condition and/or used medications, which included antiepileptic and hypnotic drugs (Table 2).

Importantly, callosotomy could be expected to determine considerable changes in brain functioning, both as an immediate consequence of surgery and in the form of slower, adaptive modifications. In our sample, complete callosotomy was performed more than 20 years before data acquisition in four patients and 8 years prior to data acquisition in the remaining one (Table 1). The relatively small sample size and the fact that patients were mostly homogeneous with respect to time passed since complete CC resection inevitably limited the possibility to investigate the role of potential time-dependent morpho-functional brain adaptations.

The effect of callosotomy on sleep spindles was investigated using a combination of hypothesis-driven and data-driven analyses, including a PCA of spindle topographic distributions.^27^^,^^42^ This analysis identified at least six main components that appeared to be preserved across HSs and patients. Of note, while the first two PCs (∼64% of the variance) appeared to reflect well-known frontal and parietal spindle ‘sources’, other PCs did not correspond to previously described patterns of spindle expression. Future studies should investigate whether the PCs observed in the present study reflect specific modes of spindle expression and/or the occurrence of particular spindle subtypes, but also whether other dimensionality reduction approaches could provide a more accurate characterization of cortical spindle involvement.

Some properties of sleep spindles, such as their frequency and density, are known to change within and across sleep cycles (e.g. Refs.^3^^,^^64^^,^^65^). A reliable investigation of overnight changes in sleep spindles was however not possible in our sample for several reasons. In fact, the presence of non-physiological activity limited the possibility to accurately distinguish N2 and N3 sleep in accordance with standard criteria. Moreover, the quality of the EEG recordings decreased significantly after ∼5 hours of sleep in most patients. Finally, during preprocessing, we removed epochs strongly affected by artifactual and non-physiological activity, thus introducing discontinuities in the recordings. Given these considerations, further investigations will be necessary to clarify whether callosotomy my differently affect particular properties of spindles as a function of their time of occurrence within cycles or across a night of sleep.

Importantly, most of the described limitations could be overcome through controlled, quantitative studies in animal models. Such investigations will therefore be critical to confirm and extend our present findings.

## Conclusions

This study investigated the morphology and topographic distribution of sleep spindles in a rare sample of patients who underwent total resection of the CC. Unlike sleep slow waves, the synchronization of sleep spindles appears to be only marginally affected by the resection of the CC, as spindle bursts continue to display a common bilateral cortical recruitment in split-brain patients. This is consistent with recent evidence indicating a strong resilience of sleep spindles to white matter damages in traumatic brain injury patients,^54^ as well as with the notion that the synchronization of sleep spindles primarily depends on the thalamus and on thalamo-cortical loops, rather than on cortico-cortical connectivity.^8^ Furthermore, we found that callosotomy is associated with a reduced spatial overlap between temporally coupled spindles and slow waves. These findings suggest that such coupled events may rely on independent synchronization mechanisms, which may act alone or in concert to promote memory formation and consolidation.^26^

## Supplementary material

Supplementary material is available at *Brain Communications* online.

## Supplementary Material

fcab108_Supplementary_Data

## Abbreviations

CC =: corpus callosum
CP =: callosotomized patient
hd-EEG =: high-density electroencephalography
HS =: healthy subject
NP =: non-callosotomized patient
PC =: principal component
PCA =: principal component analysis
