# Supplementary material for: Role of corpus callosum in sleep spindle synchronization and coupling with slow waves
Source: Brain Commun. 2021 May 25;3(2):fcab108. doi: 10.1093/braincomms/fcab108 (PMC8215432; doi:10.1093/braincomms/fcab108)
Supplement: fcab108_Supplementary_Data [file fcab108_supplementary_data.pdf]

Supplementary Material for

Role of corpus callosum in sleep spindle synchronization and coupling with slow waves

Giulio Bernardi [1 §], Giulia Avvenuti [1 §], Jacinthe Cataldi [2], Simona Lattanzi [3], Emiliano Ricciardi [1], Gabriele Polonara [4], Mauro Silvestrini [3], Francesca Siclari [2], Mara Fabri [3], Michele Bellesi [5,6]

[1] Molecular Mind Laboratory, IMT School for Advanced Studies, Lucca, Italy
[2] Center for Investigation and Research on Sleep, Lausanne University Hospital, Lausanne, Switzerland
[3] Department of Experimental and Clinical Medicine, Marche Polytechnic University, Ancona, Italy
[4] Department of Odontostomatologic and Specialized Clinical Sciences, Marche Polytechnic University, Ancona, Italy
[5] School of Bioscience and Veterinary Medicine, University of Camerino, Camerino, Italy
[6] School of Physiology, Pharmacology & Neuroscience, University of Bristol, Bristol, UK
[§] Equal contribution

Figure 1

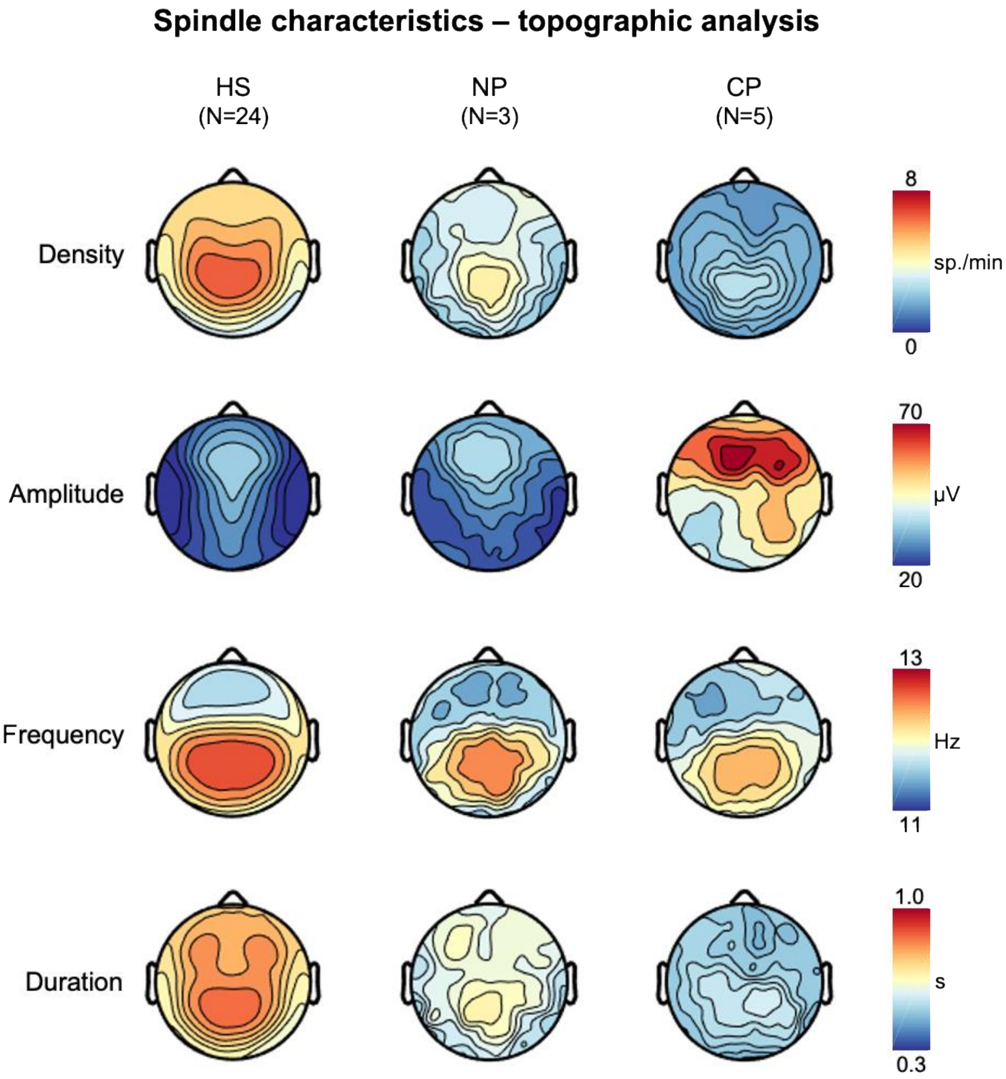

Figure 1. Properties of sleep spindles detected in individual electrodes. For analyses described in the main text, sleep spindles were detected using an approach aimed at obtaining a single time-reference for spindles occurring across different electrodes/areas. In contrast to classical approaches in which spindles are detected and analyzed independently for each electrode, the present method allows to evaluate the relative scalp distribution of each identified spindle. In order to facilitate the comparison between our approach and classical detection methods, the analysis of spindle properties was repeated using a channel-wise detection approach. Specifically, spindles were detected in individual channels (instead of multi-channel ROIs) using the same algorithm described in the main text. Then spindle density (spindles/min; first row), amplitude (µV; second row), frequency (Hz; third row) and duration (seconds; fourth row) were computed for each channel and averaged across subjects of the HS (first column), NP (second column) and CP (third column) groups.

Figure 2

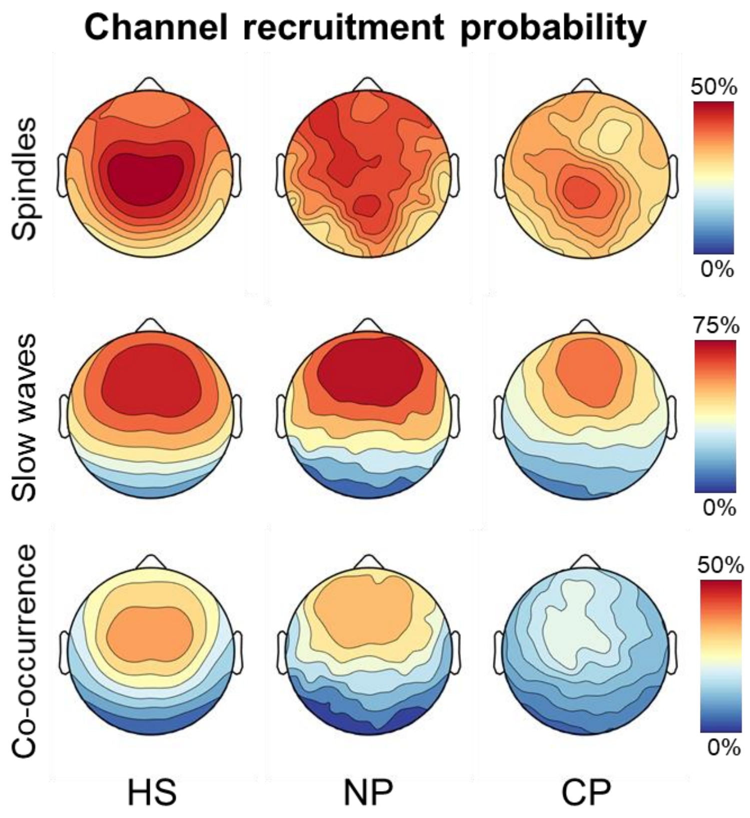

Figure 2. Channel recruitment probability for spindles (first row), slow waves (second row) and coupled spindles and slow waves (last row). The color assigned to each channel reflects the probability for that specific channel to be recruited during one of the described events.
